# Comorbid connective tissue diseases and autoantibodies in lymphangioleiomyomatosis: a retrospective cohort study

**DOI:** 10.1186/s13023-018-0933-0

**Published:** 2018-10-20

**Authors:** Shinji Futami, Toru Arai, Masaki Hirose, Chikatoshi Sugimoto, Naoya Ikegami, Masanori Akira, Takahiko Kasai, Masanori Kitaichi, Seiji Hayashi, Yoshikazu Inoue

**Affiliations:** 1 Department of Internal Medicine, National Hospital Organization Kinki-Chuo Chest Medical Centre, Sakai City, Osaka, Japan; 2 Clinical Research Centre, National Hospital Organization Kinki-Chuo Chest Medical Centre, 1180 Nagasone-cho, Kita-ku, Sakai City, Osaka, 591-8555 Japan; 3 Department of Radiology, National Hospital Organization Kinki-Chuo Chest Medical Centre, Sakai City, Osaka, Japan; 4 Department of Laboratory Medicine and Pathology, National Hospital Organization Kinki-Chuo Chest Medical Centre, Sakai City, Osaka, Japan; 5 Department of Laboratory Medicine and Pathology, National Hospital Organization Minami Wakayama Medical Centre, Tanabe City, Wakayama Japan

**Keywords:** Lymphangioleiomyomatosis, Connective tissue disease, Autoantibodies, Sjögren’s syndrome, Systemic lupus erythematosus, Rheumatoid arthritis, Antiphospholipid antibody syndrome, Comorbidity

## Abstract

**Background:**

Lymphangioleiomyomatosis (LAM) and connective tissue diseases (CTDs) occur more frequently among women than men. We investigated the frequency of comorbid CTD and positive serum autoantibody findings in patients with LAM.

**Methods:**

A total of 152 patients with LAM were prospectively and consecutively registered in the National Hospital Organization Kinki-Chuo Chest Medical Centre cohort. The clinical data were retrospectively analysed, and patients were categorised into the following three groups: a CTD group, a non-CTD-autoantibody-positive group, and a non-CTD-autoantibody-negative group.

**Results:**

All patients were women. We identified five patients with comorbid CTDs (3.3%): Sjögren’s syndrome (SjS) (*n* = 3), systemic lupus erythematosus (*n* = 1), and rheumatoid arthritis (n = 1). One patient with SjS was also diagnosed with antiphospholipid antibody syndrome. The positive rate for anti nuclear antibody was 31.5% and 6.9% at dilution of 1:40 or higher, and those of 1:160 or higher, respectively.  It tended to be lower in patients with LAM than in healthy women. The positive rate for anti-SS-A and anti-SS-B antibody was 7.9% and 1.8%, respectively. No significant differences in age, type of LAM, smoking status, serum vascular endothelial growth factor D level, respiratory function, treatment, or prognosis were observed among the three groups.

**Conclusions:**

Comorbid CTDs, especially SjS, in LAM patients should be considered.

## Background

Lymphangioleiomyomatosis (LAM) is a rare cystic lung disease caused by infiltration of smooth muscle-like cells (LAM cells) into the lungs via the circulatory and lymphatic systems [1]. LAM is almost exclusively observed among women, especially those of child-bearing age. In Japan, the prevalence rate of LAM is approximately 1.2–2.5 per million individuals [2]. As cystic lung disease can occur in various forms (e.g., chronic obstructive pulmonary disease, pulmonary Langerhans cell histiocytosis, Birt-Hogg-Dubë syndrome, and Sjögren’s syndrome [SjS]) [3, 4], a differential diagnosis for other cystic diseases should be made in cases of suspected LAM.

Moreover, previous studies have reported that morbidity due to connective tissue diseases (CTDs), including SjS, is higher among women than men [5], and that women are more frequently positive for specific CTD autoantibodies than men [6]. Indeed, some studies have reported that complications such as SjS [7] or systemic lupus erythematosus (SLE) can occur in patients with LAM [8]. However, no studies have systematically evaluated the proportion of patients with LAM and CTDs who test positive for serum autoantibodies, or whether such results influence morbidity. Therefore, the present study aimed to identify the frequency of comorbid CTDs in patients with LAM, describe the clinical features of such conditions in detail, and determine the proportion of patients with LAM testing positive for CTD serum autoantibodies.

## Methods

### Data source and study population

We obtained written informed consent from all participants prior to prospective assignment to the cohort, data collection, and serum collection (approval number: 365). The present study was also approved by the Institutional Review Board of the Kinki-Chuo Chest Medical Centre, Sakai City, Osaka, Japan (KCCMC; approval number: 531).

A total of 152 consecutive patients with LAM (131 with sporadic LAM and 21 with tuberous sclerosis complex [TSC]) who had been pathologically (*n* = 114) or clinically (*n* = 38) diagnosed at the KCCMC between January 1991 and October 2016 were initially included in the study. Further analysis revealed that five Japanese patients exhibited comorbid CTDs at the time of LAM diagnosis or during the clinical course of the disease.

Patients with LAM were divided into three groups, as follows: LAM complicated by CTD (CTD group), autoantibody-positive LAM not complicated by CTD (non-CTD-autoantibody-positive group), and autoantibody-negative LAM not complicated by CTD (non-CTD-autoantibody-negative group). The CTD group included patients diagnosed with SjS, SLE, rheumatoid arthritis (RA), or antiphospholipid antibody syndrome (APS) in accordance with the American College of Rheumatology criteria or Sydney APS Classification Criteria [9–13]. The non-CTD-autoantibody-positive group included patients who did not fulfil the diagnostic criteria for CTDs yet tested positive for one of the autoantibodies mentioned in the “*Measurement of autoantibodies*” section. The remaining patients were included in the non-CTD-autoantibody-negative group.

### Diagnosis of LAM

All diagnoses were based on the presence of multiple, bilateral cystic shadows compatible with LAM on high-resolution computed tomography (HRCT) images, and at least one of the following criteria: confirmation of LAM cells in biopsy specimens; a serum vascular endothelial growth factor D (VEGF-D) level > 800 pg/mL; or clinical findings consistent with LAM, including a chylous pleural effusion, retroperitoneal lymphangioleiomyoma, renal angiomyolipoma, or an existing diagnosis of TSC [14–20].

### Data acquisition

Data regarding age, gender, ethnicity, type of LAM, smoking status, respiratory function, clinical symptoms, diagnosis, treatments, laboratory findings at diagnosis, comorbid CTDs, and prognosis were collected from the medical records.

### Measurement of autoantibodies

Levels of the following serum autoantibodies were measured using the fluorescent antibody technique, latex coagulating nephelometry, enzyme immunoassay, or chemiluminescent immunoassay: anti-nuclear antibody (ANA) of 1:160 or higher, rheumatoid factor (RF), anti-Ro (SS-A) antibody, anti-La (SS-B) antibody, anti-neutrophil cytoplasmic antibody (ANCA), anti-double stranded DNA (dsDNA) antibody, anti-topoisomerase (Scl-70) antibody, anti-centromere antibody, anti-U1-ribonucleoprotein (RNP) antibody, anti-Smith (Sm) antibody, anti-cyclic citrullinated peptide (CCP) antibody, anti-aminoacyl-tRNA synthetase (ARS) antibody, and anti-histidyl-tRNA synthetase (Jo-1) antibody. We considered serum ANA to be positive at 1:160 or higher, because there is little pathological significance at low titre. However, we also present the data of patients with ANA of 1:40 or higher.

### Measurement of VEGF-D

Serum levels of VEGF-D were measured via enzyme-linked immunosorbent assay (ELISA) using a commercially available VEGF-D human ELISA kit from R&D Systems (Minneapolis, MN, USA). VEGF-D levels of 800 pg/mL or higher were considered diagnostic, based on methods utilised in previous studies [16, 19].

### Pulmonary function test and HRCT

Lung function tests were performed using a CHESTAC-8800™ or − 8900™ system (CHEST M.I., Inc., Bunkyo-ku, Tokyo, Japan), in accordance with the recommendations of the American Thoracic Society and European Respiratory Society [21]. The diffusing capacity of the lungs for carbon monoxide (DL_CO_) was measured using the single-breath method. All HRCT examinations were performed using a 16-channel multi-detector CT scanner (HiSpeed Ultra 16, GE Healthcare, Little Chalfont, UK).

### Prognosis

Prognosis was defined based on the time of death or lung transplantation. Patients were divided into the following two groups: transplantation/death and alive without transplantation.

### Control data

Control data for autoantibodies were obtained from previous studies involving healthy Japanese individuals [6, 22, 23].

### Statistical analysis

Continuous variables were analysed using Student’s *t*-tests or Mann-Whitney *U*-tests, depending on the normality of the data distribution. Nominal variables were analysed using Fisher’s exact tests or chi-square tests. A *P*-value of less than 0.05 was considered statistically significant.

All statistical analyses were performed using EZR Version 1.32 (Saitama Medical Centre, Jichi Medical University, Saitama, Japan), which is a graphical user interface for R (The R Foundation for Statistical Computing, Vienna, Austria). EZR is a modified version of R commander designed to add statistical functions frequently used in biostatistics [24].

## Results

### Patients with comorbid LAM and CTDs

Among the 152 total patients with LAM, five (3.3%) were diagnosed with CTDs: SjS (*n* = 3), SLE (*n* = 1), and RA (n = 1). One patient with SjS was also diagnosed with APS. All five patients were Japanese. Patient 1 underwent lung transplantation, while Patient 2 underwent treatment with sirolimus. CTDs were well controlled in all patients, with the exception of Patient 4.

### Serum autoantibodies among the 152 patients with LAM

#### Patient demographics

All 152 patients with LAM were women (Japanese, *n* = 150; Chinese, *n* = 2), with a median age of 40 years. No significant differences in age, type of LAM, smoking status, serum VEGF-D level, respiratory function, treatment, or prognosis were observed among the three groups (Table 1).

**Table 1:** Characteristics of the 152 patients with LAM

	All	CTD	Autoantibody positive *	Autoantibody negative *	*p*-value †
Number of patients	152	5	33	114	–
Sex					
Male / Female	0 / 152	0 / 5	0 / 33	0 / 114	1.00 ‡
Age at diagnosis, years §	40 (34–47)	48 (44–49)	42 (35–46)	38 (33–47)	0.22 ||
Type of LAM					
Sporadic /TSC	131 / 21	5 / 0	31 / 2	95 / 19	0.10 ‡
Smoking history					
Current / Ex / Never	6 / 29 / 117	0 / 0 / 5	0 / 6 / 27	6 / 23 / 85	0.40 ‡
Serum VEGF-D, pg/ml §	1652 (729–3183)	1413 (1048–1912)	1641 (615–3028)	1664 (762–3433)	0.53 ||
Respiratory function, %					
%FVC §	99.4 (85.3–113.0)	91.6 (87.2–95.1)	99.7 (76.4–118.0)	100.0 (85.4–111.6)	0.66 ¶
%FEV_1_ §	79.4 (61.5–105.7)	77.3 (72.8–86.0)	80.3 (56.5–107.8)	79.4 (63.0–104.2)	0.89¶
%DLco §	60.6 (39.6–82.5)	59.1 (35.1–71.2)	55.1 (38.2–74.0)	62.2 (41.9–84.7)	0.31 ||
Treatment					
mTOR inhibitor + anti-oestrogen therapy	10	0	4	6	0.51 ‡
mTOR inhibitor only	45	1	10	34	
Anti-oestrogen therapy only **	10	0	1	9	
No treatment	87	4	18	65	
Prognosis					
Transplantation / dead	9 / 9	1 / 0	1 / 3	7 / 6	1.00 ‡
Alive without transplantation	135	4	30	101	

* Autoantibody: anti-nuclear antibody 1:160 or higher, rheumatoid factor, anti-Ro antibody, anti-La antibody, anti-neutrophil cytoplasmic antibody, anti-double stranded DNA antibody, anti-topoisomerase antibody, anti-centromere antibody, anti-U1-ribonucleoprotein antibody, anti-Smith antibody, anti-cyclic citrullinated peptide antibody, anti-aminoacyl-tRNA synthetase antibody, and anti-histidyl-tRNA synthetase antibody

^†^ *p*-value is calculated between CTD plus autoantibody-positive and autoantibody-negative groups

^‡^ Fisher’s exact test, ^§^ Median (interquartile range), ^||^ Mann-Whitney *U*-test, ¶ Student’s *t*-test

** Anti-oestrogen therapy: gonadotropin-releasing hormone (GnRH) analogue (*n* = 15), and/or anti-oestrogen drug (*n* = 3), and/or pro-gestational drug (*n* = 4), or ovariectomy (*n* = 1), or radiation therapy to ovary (*n* = 1)

Abbreviations: *LAM* lymphangioleiomyomatosis, *CTD* connective tissue diseases, *TSC* tuberous sclerosis complex, *VEGF-D* vascular endothelial growth factor-D, *%FVC* percent predicted forced vital capacity, *%FEV*_*1*_ percent predicted forced expiratory volume in 1 s, *%DLco* percent predicted diffusing capacity of the lung carbon monoxide, *mTOR* mammalian target of rapamycin

#### Proportion of patients with LAM testing positive for autoantibodies

At dilutions of 1:40 or higher, serum ANA was positive in 31.5% of patients, and homogeneous, speckled, and nucleolar patterns were observed in 21.5%, 24.6%, and 3.1% of patients, respectively. At dilutions of 1:160 or higher, serum ANA was positive in 6.9% of patients, and homogeneous and speckled patterns were observed in 3.8% of patients, respectively. Positive rates for RF, anti-SS-A antibody, anti-SS-B antibody, and anti-dsDNA antibody were 13.1%, 7.9%, 1.8%, and 4.9%, respectively.

Relative to healthy women, patients with LAM exhibited a lower positive rate for ANA at dilutions of 1:40 or higher. For ANA dilutions of 1:160 or higher, the ANA-positive rate tended to be lower in patients with LAM than in healthy controls (Table 2) [6, 22, 23]. More than 70% of participants in the present study were in their 30s and 40s, while patients in this age bracket accounted for approximately 17% of participants in previous studies (Table 3) [6, 22]. No significant differences in positive rates for disease-specific autoantibodies were observed between patients with LAM testing positive for ANA and healthy women (Table 4) [22]. In addition, 14.7% and 2.9% of patients in the ANA-positive group tested positive for anti-SS-A and anti-SS-B antibodies, respectively.

**Table 2:** Comparison of the ANA-positive rate between 152 patients with LAM and healthy controls

	Country	Year	Participants	Number of subjects	ANA 1:40 or higher	*p*-value	ANA 1:160 or higher	*p*-value
Our study	Japan	2016	Patients with LAM	152	41/130 (31.5%)	–	9/130 (6.9%)	–
Hayashi [6]	Japan	2001	Healthy women	257	89/257 (34.6%)	0.62 *	34/257 (13.2%)	0.09 *
Hayashi [22]	Japan	2008	Healthy women	1409	446/1409 (31.7%)	1.0 *	174/1409 (12.3%)	0.09 *
Asanuma [23]	Japan	1997	Healthy people	113	51/113 (45.1%)	0.04 *	11/113 (9.7%)	0.57 *

* Chi-square test

Abbreviations: *ANA* anti-nuclear antibody, *LAM* lymphangioleiomyomatosis

**Table 3:** Age distribution in the 152 female patients with LAM and healthy women in previous studies

Age	20s	30s	40s	50s	60s	70s	80s	Total	*p*-value *
Our study	16 (10.5%)	59 (38.8%)	55 (36.2%)	14 (9.2%)	7 (4.6%)	1 (0.7%)	0 (0.0%)	152	–
Hayashi [6]	43 (16.7%)	45 (17.5%)	45 (17.5%)	45 (17.5%)	45 (17.5%)	34 (13.2%)	0 (0.0%)	257	< 0.01 †
Hayashi [22]	178 (12.6%)	245 (17.3%)	232 (16.5%)	202 (14.3%)	308 (21.9%)	209 (14.8%)	35 (2.5%)	1409	< 0.01 †

The percentage in parentheses represents the proportion in all cases

In a previous study, those in their 20s had the highest ANA-positive rate (16.3%), followed by those in their 60s (14.9%), 30s (13.1%), 50s (11.9%), 70s (10.5%), and 40s (9.5%) for dilutions of 1:160 or higher. For dilutions of 1:40 or higher, healthy women in their 20s had the highest ANA-positive rate (39.9%), followed by those in their 30s (33.1%), 50s (31.2%), 60s (30.8%), 70s (30.1%), and 40s (28.4%) for dilutions of 1:40 or higher [22]

^*^p-values were calculated for the distribution of age between this study and a previous study [6, 22]

^†^Mann-Whitney *U*-test

Abbreviations: *ANA* anti-nuclear antibody

**Table 4:** Positive rates for disease-specific antibodies in patients with LAM testing positive for ANA and controls^a^

	Our study	Hayashi [22]	*p*-value
Participants	Patients with LAM	Healthy women	
Number of Participants	41 †	446	–
Anti-RNP antibody	6.5% (2/31)	2.2% (10/446)	0.18 ‡
Anti-Sm antibody	0% (0/31)	0% (0/446)	1.0 ‡
Anti-SS-A antibody	14.7% (5/34)	11.2% (50/446)	0.57 ‡
Anti-SS-B antibody	2.9% (1/34)	0.90% (4/446)	0.31 ‡
Anti-Scl-70 antibody	0% (0/32)	0% (0/446)	1.0 ‡
Anti-Jo-1 antibody	0% (0/30)	0% (0/446)	1.0 ‡
Anti-centromere antibody	0% (0/19)	5.8% (26/446)	0.62 ‡
Anti-dsDNA antibody	6.5% (2/31)	1.3% (6/446)	0.09 ‡

^a^In healthy subjects, the positive rate of the disease-specific antibodies was only analysed for positive cases of ANA in the previous study [22]. Thus, only cases with positive ANA at dilutions of 1:40 or higher were analysed

^†^Forty-one patients with LAM were ANA-positive at dilutions of 1:40 or higher in this study

^‡^Fisher’s exact test

Abbreviations: *ANA* anti-nuclear antibody, *RNP* U1-ribonucleoprotein, *SS-A* Ro, *SS-B* La, *Scl-70* topoisomerase, *Jo-1* histidyl-tRNA synthetase, *dsDNA* double-stranded DNA

There were no significant differences in survival rate among the three groups: Four patients (80.0%) remained alive without transplantation in the CTD group, along with 30 patients (90.9%) in the non-CTD-autoantibody-positive group and 101 patients (88.6%) in the non-CTD-autoantibody-negative group.

### Case series of comorbid CTD in patients with LAM

#### Patient 1

A 38-year-old Japanese woman with no history of smoking was referred to our institution for cough and dyspnoea on exertion. She had been diagnosed with sporadic LAM via a surgical lung biopsy (SLB) 2 months prior to her first visit to our institution. She had a medical history of stillbirth. Schirmer test and serum anti-SS-A antibody test results were both positive. At the age of 35 years, she was diagnosed with SjS in accordance with the 2012 American College of Rheumatology Criteria [9]. She was also diagnosed with APS in accordance with the 2006 Sydney APS Classification Criteria [10]. At the time of LAM diagnosis, her levels of serum autoimmune antibodies were as follows: RF, 68 IU/mL; anti-dsDNA antibody, 24 IU/mL; anti-cardiolipin antibody, 11 IU/mL; anti-SS-A antibody > 500 U/mL; and anti-SS-B antibody < 7.0 U/mL.

**Fig. 1:**
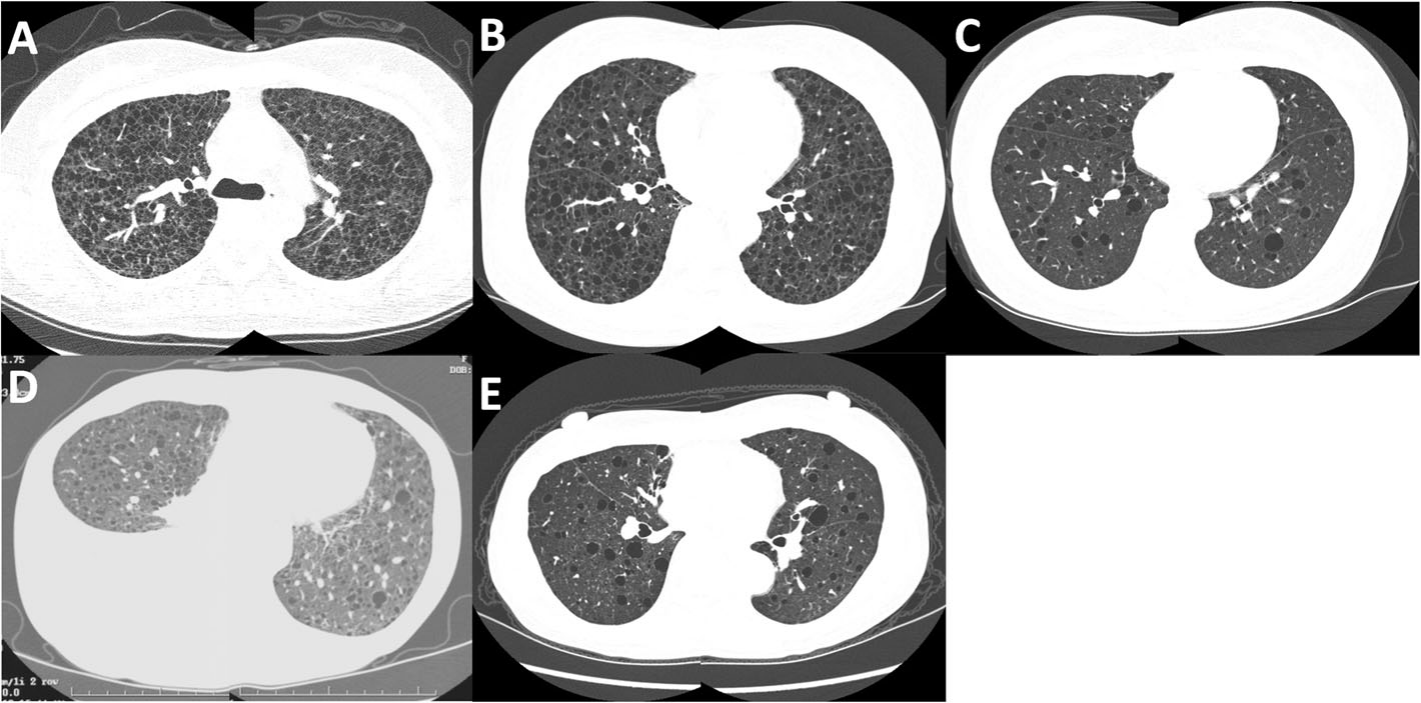
Chest CT findings in five patients with lymphangioleiomyomatosis (LAM) and comorbid connective tissue diseases. All five patients exhibited multiple, diffuse, thin-walled cystic lesions. **a** Patient 1: A 38-year-old women with LAM, Sjögren’s syndrome, and antiphospholipid antibody syndrome. **b** Patient 2: A 61-year-old patient with LAM and comorbid Sjögren’s syndrome. **c** Patient 3: A 48-year-old patient with LAM and comorbid Sjögren’s syndrome. **d** Patient 4: A 44-year-old patient with LAM and comorbid rheumatoid arthritis. The examinations revealed right pleural effusion. **e** Patient 5: A 49-year-old patient with LAM and comorbid systemic lupus erythematosus

**Fig. 2:**
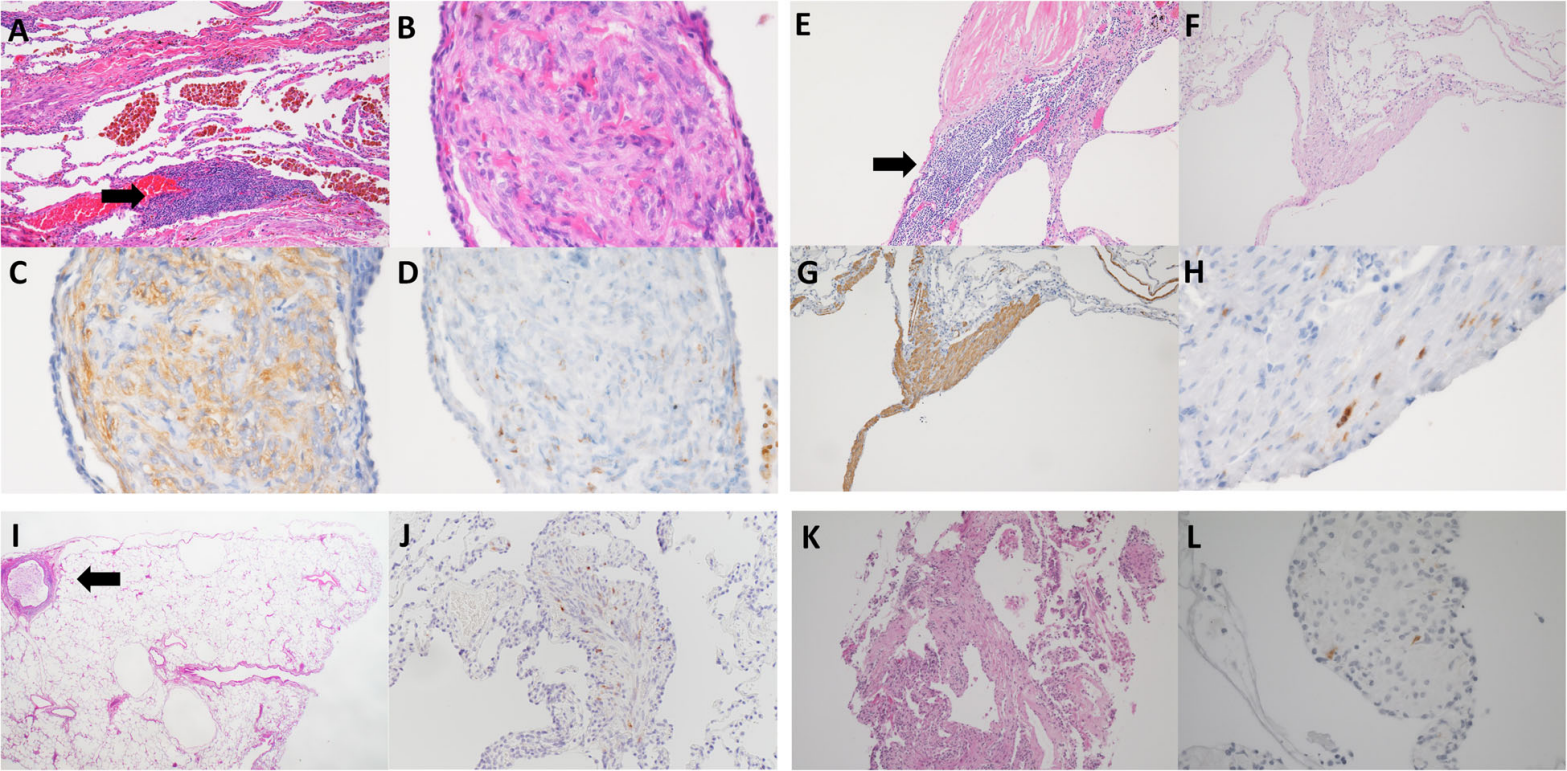
Pathological findings in patients with lymphangioleiomyomatosis (LAM) and comorbid connective tissue diseases (Cases 1–4). **a-d** Photomicrographs of surgical lung biopsy (SLB) in Patient 1. **a, b** Haematoxylin and eosin (**H**&**E**) staining was observed at a magnification of × 10 and × 40, respectively. Clumps of spindle cells with eosinophilic cytoplasm were noted in the lung interstitium and regarded as LAM cell nests (central right, lower left) (**b**). The lung interstitium around the small blood vessels exhibited a small lymphoid follicle (lymphoid cell aggregates) (arrow). **c, d** Alpha-smooth muscle actin (αSMA) and human melanoma black-45 (HMB45) immunostaining results were positive in LAM cell nests (magnification, × 40). **e-h** Photomicrographs of SLB in Patient 2. (**e**) **H**&**E** staining revealed lymphoid cell aggregate (arrow) and focal fibrotic lesions in the wall of a cystic lesion (7 × 14 mm) as well as proliferation of LAM cells (magnification, × 10). **f**, **H**&**E** staining revealed another cystic lesion and alpha-MA-positive LAM cell nest in the wall (alpha-SMA, not shown) (magnification, × 10). **g, h** A LAM cell nest testing positive for αSMA and HMB45 antibodies (magnification, × 40). **i, j** Photomicrographs of SLB in Patient 3. **i**, **H**&**E** staining showing infiltration of lymphoid cells into the wall of a membranous bronchiole (arrow) and two cystic lesions measuring 1.5 × 1 mm and 1.7 × 1.2 mm due to LAM (magnification, × 2). **j** Positive HMB45 staining was observed in a LAM cell nest in the lower right area of **i** (magnification, × 40). **k, l** Photomicrographs of transbronchial lung biopsy in Patient 4. (K) H&E staining revealed a LAM cell population (central area) with eosinophilic cytoplasm, which tested positive for oestrogen receptor (ER) and progesterone receptor (PgR), in the wall of a D2–40 positive-cell lined lymphatic vessel measuring 200 μm in diameter (central lower area) (magnification, × 10) (ER, PgR, and D2–40, not shown). **l** Another LAM cell nest testing positive for HMB45 following transbronchial biopsy (magnification, × 40)

Diffuse, thin-walled cystic lesions were observed on HRCT (Fig. 1a). An SLB was performed at segment 6 of the right lower lobe. The lung tissues exhibited spindle cell nests in the interstitium. Further examination revealed that these LAM cell nests were positive for alpha-smooth muscle actin (αSMA), human melanoma black-45 (HMB45), oestrogen receptors, and progesterone receptors. Formation of lymphoid follicles (lymphoid cell aggregates) was observed in multiple areas of lung tissue (Fig. 2a-d).

The patient received no medication for LAM or decreases in respiratory function. At the initial and 6-month follow-up visits, her percent predicted forced vital capacity (%FVC) values were 87.2% and 82.0%, her percent predicted forced expiratory volume in 1 s (%FEV_1_) values were 49.1% and 46.5%, and her percent predicted diffusing capacity of the lung for carbon monoxide (%DLco) values were 26.0% and 18.9%, respectively. She underwent lung transplantation 51 months after the first visit to our institution.

#### Patient 2

A 61-year-old Japanese woman with no history of smoking was referred to our institution for dyspnoea on exertion. The patient had been diagnosed with sporadic LAM via SLB 2 months prior to her first visit to our institution. She had a medical history of pneumothorax. Autoimmune antibody tests were negative at the initial visit, although she tested positive for anti-SS-A antibody (28.4 U/mL) 38 months after the first visit. She received a diagnosis of SjS based on 2012 American College of Rheumatology Criteria [9].

HRCT revealed diffuse, thin-walled cystic lesions (Fig. 1b). SLB was performed from the lingular segments of left upper lobe and left lower lobe. Proliferation of LAM cells was observed in the interstitium, while immunostaining experiments revealed that the LAM cell nests were positive for αSMA and HMB45. Cystic lesions were observed within the lung tissue, along with some lymphoid follicles and lymphoid cell infiltration in the peribronchiolar regions (Fig. 2e-h).

Sirolimus treatment was initiated 30 months after the first visit. The patient remained alive at the 6-year follow-up, with no further decreases in pulmonary function (%FVC: 141.2%, %FEV_1_: 101.0%, %DLco: 61.2%). Administration of sirolimus did not affect the course of SjS.

#### Patient 3

A 48-year-old Japanese woman with no history of smoking was referred to our institution due to the presence of abnormal shadows on chest radiographs. She was diagnosed with sporadic LAM via a SLB 4 months after her first visit to our institution. She had a medical history of uterine myoma and diffuse goiter. Lip biopsy revealed infiltration of lymphocytic cells, and serum anti-SS-A antibody test results were positive (12.1 U/mL). The patient was diagnosed with SjS in accordance with the 2012 American College of Rheumatology Criteria [9].

HRCT revealed diffuse, thin-walled cystic lesions (Fig. 1c). Although a transbronchial lung biopsy (TBLB) was performed, it did not lead to the diagnosis of LAM. SLB was performed at segments 4 and 8 of the right lung. Cystic lesions of up to 8 × 6 mm in size were observed within the lung tissues, along with proliferation of LAM cells in the interstitium. Immunostaining experiments revealed that LAM cells were positive for HMB45, αSMA, and oestrogen receptors (Fig. 2i, j). Lymphoid follicles with germinal centres in the walls of membranous bronchioles (500 × 500 μm) and chronic interstitial pneumonia with a subpleural focus were observed within at 2.0 × 2.5 mm area using a microscope. Honeycombing and band-like infiltration of lymphoid cells was observed within a visceral pleura measuring 150 × 2500 μm.

She received no medication for LAM, and no further decreases in respiratory function were observed at the 8-month follow-up (%FVC: 98.8%, %FEV_1_: 83.4%, %DLco: 117.5%).

#### Patient 4

A 44-year-old Japanese woman with no history of smoking was referred to our institution for dyspnoea at rest and subsequently diagnosed with sporadic LAM via TBLB. She had a medical history of RA, which was diagnosed in accordance with 1987 American College of Rheumatology Criteria [11] and treated with bucillamine, methylprednisolone, and salazosulfapyridine. Levels of serum autoimmune antibodies were as follows: RF 46 IU/mL, ANA 1:160 (speckled).

HRCT revealed diffuse, thin-walled cystic lesions and right pleural effusion (chylothorax) (Fig. 1d). TBLB was done performed in the upper and lower portions of the right lung. LAM cell populations with eosinophilic cytoplasm encompassing the wall of the dilated lymphatic vessel were observed in TBLB specimens. Immunostaining experiments revealed that these LAM cells were positive for αSMA, oestrogen receptor, progesterone receptor, and HMB45 (Fig. 2k, l).

She received no medication for LAM. No long-term follow-up data regarding respiratory function and LAM were obtained due to her difficulty in visiting the hospital. Her initial values were as follows: %FVC: 79 8%; %FEV_1_: 72.8%; %DLco: 35.1%. Her survival one month after the first visit was confirmed.

#### Patient 5

A 49-year-old Japanese woman with no history of smoking was referred to our institution for dyspnoea at rest. She was diagnosed with sporadic LAM based on histological examination of a retroperitoneal tumour (lymphangioleiomyoma) 4 months prior to the first visit. She had a medical history of SLE, uterine myoma, and pneumothorax. SLE was associated with pleurisy, proteinuria, and psychosis. She was diagnosed with SLE in accordance with the updated 1997 American College of Rheumatology Criteria [12, 13]. SLE was treated with prednisolone. Serum levels of autoimmune antibodies were as follows: ANA 1:80 (homogeneous, speckled); anti-dsDNA antibody, 7.6 U/mL.

HRCT revealed diffuse, thin-walled cystic lesions (Fig. 1e). A retroperitoneal tumour measuring 12.5 × 8.4 cm in size was resected. Histological examination revealed a lymphangioleiomyoma testing positive for αSMA and HMB45.

She received no medication for LAM and was treated with prednisolone (5 mg/day) for SLE. However, no decreases in pulmonary function were observed during the 12 years between her initial and most recent visit (%FVC: 102.0%, %FEV_1_: 85.4%, %DLco: 86.6%).

## Discussion

The present study is the first large-scale investigation of comorbid CTD in patients with LAM. We identified a total of five patients with comorbid CTD among the 152 included patients with LAM. In our study, the prevalence rates of SjS, APS, RA, and SLE were 1.97%, 0.66%, 0.66%, and 0.66%, respectively. Current estimates of SjS, SLE, and RA prevalence are 0.05–0.7% [25], 29 per million [26], and 0.41% [27], respectively. The prevalence of APS is uncertain, although the frequency of antiphospholipid antibodies in has been reported as 1–5.6% in healthy controls [28]. These findings indicate that SLE, SjS, RA, and APS may be equally or more frequently observed in LAM than in the general population. However, it is necessary to pay attention to the possibility that both LAM and CTD may incidentally happen in same patients because healthy individuals also exhibited a high positive rate of ANA, anti-SS-A, and anti-SS-B. (Tables 2, 4).

There were no significant differences in prognosis among the three groups in our study; thus, there is no evidence to support the notion that comorbid CTDs affect the progression and prognosis of LAM. Patient 2 was diagnosed with SjS during a follow-up visit regarding LAM. Such findings indicate that patients with LAM should be monitored for signs of CTD.

Loss of function mutations in *TSC1* and *TSC2* have been broadly detected in pulmonary LAM cells: These mutations activate mammalian target of rapamycin (mTOR) protein kinases [29]. Recent studies have reported that the mTOR pathway is associated with SLE, APS, and RA. The activity of mTOR increases in human SLE [30], and activation of mTOR plays a pivotal role in the abnormal activation of T- and B-cells in SLE [31]. In cultured vascular endothelial cells, IgG antibodies from patients with APS stimulate the mTOR complex via the phosphatidylinositol 3-kinase (PI3K)-AKT pathway [32]. Furthermore, mTOR signalling is active in the synovial membrane of patients with RA. Knockout of PI3Kγ, a protein kinase upstream of mTOR, diminishes tumour necrosis factor-driven cartilage damage [33]. Previous studies have also reported that activation of interferon alpha (IFNα), B-cell activating factor (BAFF), and antibodies to muscarinic acetylcholine receptors is associated with the development of SjS [34]; and that sirolimus inhibits BAFF-stimulated cell proliferation [35]. Thus, SjS may be associated with the mTOR pathway. It is not certain whether mTOR overactivation increase the risk of CTD remains to be answered, but LAM may be associated with the occurrence of CTDs such as SjS, SLE, RA, and APS.

The present study is also the first large-scale investigation of serum autoantibody levels in patients with LAM. In the present study, the positive rate for ANA tended to be lower in patients with LAM than in the general population (Table 2). However, the distribution of age differed between our study and previous studies [6, 22]. Patients with LAM in our study were more frequently in their 30s and 40s (Table 3). Thus, the difference in age distribution may have affected the results.

Our study possesses some limitations of note. First, serum levels of autoantibodies were not measured in all patients with LAM, and not all patients underwent physical examination by a rheumatologist. Furthermore, not all patients underwent routine follow-up at our hospital, indicating that more patients may have had comorbid CTD. Second, this retrospective study was performed at a single institution. Third, the three groups are very imbalanced in size (only five patients were included in CTD group), which makes any statistical comparisons doubtful. However, LAM is a rare lung disease, with a relatively low rate of CTD comorbidity, making prospective studies rather difficult. Future multicentre studies are required to more fully elucidate the association between LAM and CTD.

## Conclusion

Our findings indicated that 31.5% and 6.9% of patients with LAM had positive ANA results at dilutions of 1:40 or higher, and those of 1:160 or higher, respectively, and 3.3% had CTDs. Comorbid CTDs, especially SjS, in LAM patients should be considered.

## Abbreviations

%DLco: Percent predicted diffusing capacity of the lung for carbon monoxide
%FEV_1_: Percent predicted forced expiratory volume in 1 second
%FVC: Percent predicted forced vital capacity
ANA: Anti-nuclear antibody
ANCA: Anti-neutrophil cytoplasmic antibody
APS: Antiphospholipid antibody syndrome
ARS: Aminoacyl-tRNA synthetase
BAFF: B-cell activating factor
CCP: Cyclic citrullinated peptide
CTDs: Connective tissue diseases
DLco: Diffusing capacity of the lung for carbon monoxide
dsDNA: Double stranded DNA
ELISA: Enzyme-linked immunosorbent assay
HMB45: Human melanoma black-45
HRCT: High-resolution computed tomography
IFNα: Interferon alpha
Jo-1: Histidyl-tRNA synthetase
KCCMC: Kinki-Chuo Chest Medical Centre
LAM: Lymphangioleiomyomatosis
mTOR: Mammalian target of rapamycin
PI3K: Phosphatidylinositol 3-kinase
RA: Rheumatoid arthritis
RF: Rheumatoid factor
RNP: U1-ribonucleoprotein
Scl-70: Topoisomerase
SjS: Sjögren’s syndrome
SLB: Surgical lung biopsy
SLE: Systemic lupus erythematosus
Sm: Smith
SS-A: Ro
SS-B: La
TBLB: Transbronchial lung biopsy
TSC: Tuberous sclerosis complex
VEGF-D: Vascular endothelial growth factor D
αSMA: Alpha-smooth muscle actin
